# Exploration of a Co-Production Approach to Developing a Walking Group with People with Huntington’s Disease

**DOI:** 10.20900/mo.20190022

**Published:** 2019-10-31

**Authors:** Una Jones, Katy Hamana, Sofia Vougioukalou, Mel Jones, Monica Busse

**Affiliations:** 1 School of Healthcare Sciences, Cardiff University, Heath Park, Cardiff CF14 4XN, UK; 2 Cardiff School of Sport and Health Sciences, Cardiff Metropolitan University, Llandaff Campus, Cardiff CF5 2YB, UK; 3 Centre for Trials Research, Cardiff University, Neuadd Merionnydd, Heath Park, Cardiff CF14 4XN, UK

**Keywords:** physical activity, walking groups, co-production, Huntington’s disease

## Abstract

**Background:**

People with Huntington’s disease (HD) struggle to maintain regular physical activity despite evidence of the benefits of exercise. This study aimed to evaluate the experiences of people who co-produced a walking group for people with HD.

**Methods:**

Three people with HD, a specialist HD advisor (sHDA), two project officers from Let’s Walk Cymru (LWC) and the research team co-produced and participated in a walking group for people with HD. A walking group for people with HD was supported weekly by LWC for eight weeks and fortnightly for a further 12 weeks. Semi-structured interviews were undertaken with three people with HD, a sHDA and two project LWC project officers. Interviews were transcribed verbatim and analysed using thematic analysis.

**Findings:**

Interviews identified six themes across participants: “organisation and planning”; “purpose of the walks”; “benefits”; “barriers”, “the group” and “the future”. People with HD enjoyed participating in the walks and reported increased confidence to be more active outside the home. All participants noted challenges including apathy, diminished planning skills, social stigma and motor problems specific to HD; people with HD perceived a lack of influence in relation to co-planning and co-execution of the walking group.

**Conclusions:**

The walking group was perceived as enjoyable, beneficial, and motivational. This is the first study to report co-production of a walking group with people with HD and the findings suggest that further research is needed to adapt models of co-production for people with a long-term complex condition.

## Introduction

Huntington’s disease (HD) is caused by an autosomal dominant gene that produces mutant huntingtin protein that is characterised by abnormally long CAG repeats. Abnormal huntingtin results in neuronal dysfunction and death, particularly of the medium spiny neurons in the striatum [1]. The motor problems associated with neuronal loss include chorea, bradykinesia and dystonia [2]. The contradictory symptoms of chorea and bradykinesia have been explained by specific loss of medium spiny neurons in the indirect pathway of movement control at the early stages of the disease leading to hyperkinesia, with loss of medium spiny neurons in the direct pathway later in disease progression leading to hypokinesia [1,3]. Motor problems are compounded by cognitive dysfunction including progressive decline in planning and the ability to acquire new psychomotor skills as well as depression and apathy [4]. The triad of motor, behavioural and cognitive impairments related to HD is therefore challenging when physiotherapists and other health professionals support people to remain as functionally independent for as long as possible.

People with HD struggle to maintain regular participation in physical activity [5] despite emerging evidence supporting the role of exercise for people with HD [6]. This systematic review demonstrated that people with HD had improved motor function, gait speed and balance as a consequence of an exercise intervention. Additionally, people with HD and their caregivers perceived benefits of exercise as increased self-confidence and independence as well as enhanced social relationships. Perceived barriers were physical factors such as poor balance, lack of motivation and cognitive impairment. Physical activity, which incorporates exercise as well as recreational activities, has general health benefits including positive effect on wellbeing, sense of achievement and relaxation and it is recommended that adults undertake 150 min of moderate intensity physical activity per week [7].

Walking at three to five miles per hour is considered a moderate intensity physical activity and walking groups have wide ranging health benefits for the general population [8]. Limited evidence exists regarding walking groups for people with HD, with just one study from The Netherlands showing that a physiotherapy led walking programme was feasible and may slow total functional decline [9].

Promotion of physical activity is integral to healthcare provision and despite evidence for the benefits of physical activity, translation into practice is limited [10]. This may be due to lack of involvement of healthcare professionals and people with health conditions in research study interventions and implementations [11]. Co-production approaches, whereby public and/or patients and healthcare professionals interacting with organisations outside of the healthcare systems [12] may be necessary for promotion and uptake of physical activity, particularly for people with functional limitations.

The triad of motor, cognitive and behavioural dysfunction in people with HD may influence their ability to undertake physical activity and this could be further hindered by lack of promotion by healthcare professionals and provision of suitable activities within the community. In order to explore ways to maintain regular participation in physical activity, this study aimed to investigate a co-productive approach including people with HD and service providers for developing a walking group for people with HD. As no previous studies have explored co-producing physical activities with people with HD, this study will provide unique information for healthcare professionals and people working in the non-profit organisations to enhance service provision.

## Methods

A qualitative descriptive approach was used in this study. This approach is recommended in healthcare environments as it provides rich descriptive content from the participants perspectives and allows the researcher to choose from a number of theoretical frameworks, sampling and data collection methods [13]. The conceptual model of healthcare service co-production described by Batalden *et al.* [12] was used as the framework for this study. This model acknowledges the blurred roles of patients and professionals as well as the blurred boundary of healthcare within the larger community and is therefore appropriate for the development of a physical activity programme supported by healthcare professionals in a community setting. Within these blurred boundaries, civil discourse, *i.e.*, effective communication; co-planning and co-execution are recognised as key elements of co-production of healthcare services.

Ethical approval, including agreement from participants for the use of pseudonyms and that data could be used in academic publications, was gained from the Cardiff University, School of Healthcare Sciences Research Ethics Committee (2016 May 17).

### Co-Planning of the Walking Group

Co-planning and civil discourse [12] underpinned a co-production event involving three people with HD, the local specialist Huntington’s Disease Association Advisor (sHDA), Let’s walk Cymru (LWC) and the research team. Specialist HDA advisors support people with HD in the community and have backgrounds in either health or social care. Let’s Walk Cymru was a Welsh Government funded initiative run by Ramblers Cymru that aimed to support develop new walking groups across Wales. Convenience sampling was used and people with HD were recruited via the sHDA. On advice from LWC, discussion topics at the co-production event included: use of closed, *i.e.*, people with HD only or open groups; information giving walks; communication methods; risk assessment; volunteer recruitment; weather implications, and advertisement of walks.

Participants agreed on weekly walks; preference for closed groups; a regular meeting place and time and that people with HD should take increasing responsibility over 12 weeks to organise and plan the walks, aligning with the principle of co-execution within the conceptual model. LWC agreed to take overall responsibility for planning, designing and leading walks and also monitoring walks for safety.

### Co-Execution of the Walking Group

Convenience sampling of three people with HD were recruited by the sHDA from 60 meetings with people with HD in South Wales and via a poster at the local HD Research and Management clinic. The minimum requirements for walkers were: ability to walk independently with or without a walking aid for 20 min; able to independently get up from the floor. The Physical Activity Readiness Questionnaire [14] was used to screen people for suitability of walking.

The walk started at 2 pm each Tuesday from a city centre location. Walks were supported by the research team, LWC and the sHDA on a weekly basis for eight weeks and consequently on a fortnightly basis for 12 weeks by LWC. LWC provided support by communicating with participants to confirm walk details; planning, leading and ensuring safety throughout the walks. The research team and sHDA walked with the participants to provide a walking group atmosphere and the research team funded the coffee stops. Lead walker training comprising both theoretical and practical aspects of health walks was made available to people with HD from LWC. Theoretical aspects included understanding the benefits of walking, organisation of paperwork, carrying out risk assessments and how to deal with difficult scenarios.

### Evaluation of Experiences of the Walking Group

Semi structured interviews, were carried out by researchers UJ and KH with people with HD, the sHDA and two project officers from LWC after the eighth walk. Interviews were held at participants home (*n* = 2) and the university (*n* = 4). Images taken during the walks were used as prompts during the interviews to elicit memories. Questions were focused around personal experiences of the walks including hopes and concerns, feelings before and during the walks, social aspects and future of the walking group. Pre-selected prompts included exploring motivation to walk, physical and emotional impact of walking and setting routines.

### Measurement of Mobility

Extent of mobility was assessed before and after eight weeks using the University of Alabama Life-Space Assessment [15], which assesses range, frequency and independence of mobility with scores ranging from 0 to 120; higher scores indicating better mobility.

### Data Analysis

Field notes were taken and interviews were audio-recorded and transcribed verbatim; participants were given pseudonyms to protect their identities. Inductive thematic analysis [16], carried out by UJ and KH, was used to identify key themes and salient concepts. This method of analysis was chosen as it allowed for rich description of data through identification and analysis of patterns. Phases of thematic analysis included familiarisation of data, generation of initial codes, searching and reviewing themes; defining and naming themes.

Familiarisation of data was followed by initial double coding. Codes were identified from the data and were generated by selecting segments of text and assigning codes to organise the data into meaningful groups. Awareness of one’s biases during data analysis reduced the likelihood that data that was only of interest from a certain point of view, were selectively screened out or included. Double coding at this stage reduced the likelihood of bias being introduced from an early stage of data analysis, promoting credibility of the findings [17]. Reflexive discussions between UJ and KH identified themes from the data. Reflexivity involved critical discussions during data collection and analysis to ensure dependability. Credibility was ensured by inductive development of themes from the data.

Values for the Life-Space assessment were computed for each level of the questionnaire by multiplying the Life-Space level (1–5), the degree of independence (2 if independent, 1.5 if equipment was used, and 1 if personal assistance was reported), and the frequency of attainment (1 = less than once a week, 2 = 1–3 times a week, 3 = 4–6 times a week, and 4 = daily). The level-specific values were summed [15]. The values were analysed descriptively due to low numbers of participants.

## Findings

Three people with HD participated in the walking group over the first eight weeks supported by the research team; two people met independently during the 12 weeks when supported fortnightly by LWC. The three individuals have not met since support was discontinued. One person with HD undertook walk leader training provided by LWC, but did not lead any walks.

The fully supported walks took place within a large city centre park, the last walk being at an outdoor national history museum. Two walks in the park had specific foci: a QR heritage trail and a visit to a castle. The pathways within both the park and the history museum were broad and well surfaced and the visit to the castle and museum included steps. The walks lasted between 50 and 110 min, which included stopping at coffee shops.

Details of living and working status and Life-Space scores for people with HD are summarised in Table 1.

Six themes were identified from the interviews: the purpose of the walks, perceived benefits, barriers, the group, organisation and planning of the walks and the future.

### Purpose of the Walks

A variety of perspectives of the purpose of the walking group were identified by people with HD, LWC (1 and 2) and sHDA. All participants thought that the initial idea of a walking group was good and achievable.

LWC1—“I think the reason people pick walking is because it is a safe activity.”

sHDA—“I really thought that people would think this is a great chance to do something together in a supportive environment.”

Additionally, the social aspect of the group was perceived as positive with Sally describing a reciprocal understanding between people with same condition:

Sally—“I understood what’s he’s coming from, he understands where I was coming from.”

John and George had more specific reasons for being involved in the walking group, both from altruistic and personal perspectives:

John—“I have to help people that’s why I got involved.”

George—“The interesting bit is a plus—going to different places.”

These perspectives reflect a common understanding of the potential benefits of the walks.

### Perceived Benefits

Benefits of the walking group were perceived as both mental and physical as well as increasing confidence. Mental health benefits reported included a better frame of mind, more energy and increased motivation afforded by walking. Prompts related to motivation and physical and emotional impact of walking elicited the detail provided by participants.

George—“I feel the walking group has motivated me to get out and meet other people …. I am normally in a better frame of mind when I finish the walks.”

Physically, people with HD felt that they had better strength and balance. One participant reported fewer falls and believed this to be a consequence of walking more.

Sally—“I've always walked, I walk every day, but my balance seems a lot better now actually. I can walk further without getting breathless, so overall I just think it boosts all of you …. No I don't fall now ….”

These benefits appeared to give people with HD more confidence.

John—“my confidence, strength, my ability to walk properly again is coming back.”

In sharing his experience of the walking group, John was clear about how the gain in confidence and ability motivated him to challenge himself in activities outside the walking group.

### Barriers

Amongst the participants, various barriers to taking part in the walking group were identified. These included general barriers that may be expected from anyone aiming to increase physical activity and those specific to the complexity of HD.

Transport to the walk location was identified as a barrier by the sHDA and people with HD.

sHDA—“a couple of people said well, if someone could pick me up [I could go] … some of them, the geography just wasn’t going to work.”

This may be applicable for any organised physical activity event, yet is compounded by the rarity of HD and therefore difficulty in finding a geographically convenient walking location. Other barriers specific to HD included the genetic and progressive nature of the condition. John did not want his daughter to come along and see someone with more advanced symptoms in the group.

John—“… thinking that’s what I am going to be like in 10 years’ time … or that's what she's going to be like in 25 years’ time.”

This was mirrored by what people told the sHDA.

sHDA—“… the thought of being with other people that are at different stages … is he worse than I am ….”

Lack of motivation, a specific issue for people with HD was reported as barrier by sHDA.

sHDA—“they [carers] can’t get them to make a cup of tea at home, they don’t think they are actually going to make them go on a walk.”

Participants also shared barriers of a general nature, e.g., financial consequences of travel, perceived negative expectations, unrealistic fitness levels required for the activity.

### The Group

The theme of the group had two sub-themes: individual needs within the group and the social aspect of being in a group. John, George and LWC identified that the pace and distance was determined by the least able member of the group.

John—“George and I are not being pushed enough…. Obviously I understand you’ve got to work to the weakest person. Unfortunately for the rest of us, got to walk at a snail’s pace.”

LWC2—“because she does need, from what I can gather she needs more support than he does.”

George—“Yeah [laughter] and when somebody wanted a rest we all just stopped and had a rest.”

LWC also identified that different people needed different support.

LWC2—“So we’ve kind of almost with John, we’d need to tailor, make what he needs to do, and then perhaps Sally and George are more easily … um … catered for within a more general setting.”

Participants referred to the positive social aspects of being in a group, with LWC commenting on how this can increase the distance walked.

John—“… I wouldn’t have minded spending time with George, that’s why I phoned him up. If he came along yesterday then I would have shown him what I think is improving me.”

LWC1—“And in actual fact they probably walk further because they’re talking and have no idea where they’re going.”

People with HD expressed their enjoyment at being part of a group and the fun that could be had being with other people.

Sally—“I think every walk there is something funny…. George and his mac, yeah that was a good memory.”

George—“I enjoy the walk more if there’s more than me there.”

Participants were in agreement that social aspects including helping each other, enjoyment and developing friendships were inherent to being part of a group. Needs of the individual impacted both positively and negatively on the group.

### Organisation and Planning of the Walks

Participants identified different aspects related to organisation of the walks, including logistics and underlying motive for the walking group. Prompts around setting of routines enabled George to identify the benefits of a consistent start point and time.

George—“I think it has helped being in the same place and time or same day and time … yeah because it’s something you just get used to.”

LWC also commented on how improvements in organisation of walks could be made:

LWC2—“I think maybe if you have small numbers again … go for a group that is already established rather than trying to develop your own.”

People with HD perceived that there were too many healthcare professionals in relation to people with HD and that the focus was not on co-production of a walking group but rather on completion of a research study.

John—“the research needs guinea pigs … I don’t think any thought went into it, there was, everybody had an idea what was going to happen and everyone else shoe-horned into it.”

George—“… lots of chiefs and a few Indians ….”

Participants recognised that organisation of the walks was important, but that this could be influenced by perceptions of the purpose of the walking group.

### The Future

All people involved in the study felt that a walking group should continue and discussed factors that could influence the sustainability of the group. Specific ways of supporting the group were suggested.

LWC2—“… you could bring the physical into the clinic … you could take them for a walk around the grounds … [we could] come into clinic and have a chat, and for people to get to know what Let’s Walk is and what they were going to expect.”

John recognised that resources are needed to support a walking group and LWC agreed that specific support was needed.

John—“I think a better way would be to … pay somebody an expense and … go with those on a one-to one basis.”

LWC1—“I think activity co-ordinators would be a good start.”

LWC identified that a walking group would need organisation, which may be problematic.

LWC2—“they [independent walking groups] would have to fill in paperwork every time so there is a few little stumbling blocks.”

It was also felt that advertisement of the group also needed to be more prominent.

Sally—“we need to advertise it more.”

LWC1—“But I think if we can advertise a monthly HD walk within the clinic, then that might be something that’s on, they’ll diary it and will come to.”

Overall, participants agreed that the walking group needed to continue but recognised that more needed to be done to ensure this, e.g., integration of walks with clinic visits, advertisement. Linked to the theme of “barriers”, engaging more people in the walking group was identified as a way forward.

## Discussion

Three people with HD took part in a regular supported walking group, developed using the Batalden *et al.* [12] conceptual model of co-production. Recruitment of people with HD to the walking group was challenging, mainly due to negative perceptions of walking in a group of people with HD due to social stigma and lack of general public awareness of HD. However, people with HD enjoyed participating in the walks as a small group and perceived physical, psychological and social benefits. Positively, these participants reported increased confidence in being more active outside the home which may explain the increase in mobility as measured by the Life-Space data. Despite the perceived benefits of the walking group, when support from the wider project team was no longer provided, the HD group members did not continue to meet.

Reasons for the walking group not continuing may relate to the complexity of HD pathology in relation to both individual ability and the process of co-production and also lack of resources. A designated walk leader that could either integrate physical activity within clinical services or organise regular walks in the community may be needed to support people with HD, as the success of the walking group in The Netherlands may be due to it being led by physiotherapists [9].

For the participants with HD in this study, the motor problems of chorea and hypokinesia were not identified as barriers to participating in the walking group, yet perceived physical benefits of improved balance and strength were acknowledged. This sample however only included those who were able to walk independently, therefore this finding would not be generalisable to the wider population of people with HD. Unrealistic fitness levels, identified by participants as a barrier, may have been a reason for people with HD with decreased functional ability due to marked chorea or hypokinesia not participating in the study.

For people with HD, the organisation and planning required to continue the walking group may be compromised by disease pathology. It became apparent via field notes that the participants with HD were not willing to take sole responsibility for these organisational roles, *i.e.*, co-execute, due to behavioural and cognitive limitations. Cognitive slowing and problems with mental flexibility, planning and initiation of action are characteristic of HD [18] and may reduce the ability to plan a safe walking route and organise starting points and times. Apathy may also impact on the ability to initiate the planning required to get to the starting point of the walk. This was identified by the sHDA, who described how carers were unable to get people with HD to make a cup of tea and therefore lack of motivation related to joining a walking group may be a barrier.

Promotion of physical activity for people with HD remains a complex and challenging area. Co-production in the provision of healthcare facilitates development of services to maximise health and well-being, yet not all people with health problems have the desire or capacity to be active partners [12]. Co-production that includes people with HD, must therefore take into consideration both the consequences of HD pathology and the interaction with others that is necessary for people with HD to engage with and participate in physical activity.

A central assumption of co-production is that a common ground is found between participants and this may inherently challenge both the role and perceived hierarchy of power of the healthcare professional [19]. All participants in this study, including the research team, had a common understanding that walking was a beneficial activity, yet people with HD saw the role of the research team, and possibly of the healthcare provider, as being in control and having the ultimate power within the walking group. Participants therefore viewed the walking group as being organised by “you” for “us”. This perceived lack of self-efficacy may be due to participants transferring the hierarchy of power in health provision to the context of the provision of the walking group by the research team. Previous research has identified that people with HD did not take ownership of an exercise programme if it was delivered to them rather than being a partner who decides the nature and course of the programme [20]. A different approach to implementing evidence based practice from a researcher’s perspective may also be required. The traditional assumption that research is produced, packaged and made accessible to non-academics is challenged in co-production, when power and perceptions require careful negotiation [21]. A longer process of co-planning the walks may have been beneficial in this study to develop trust and explore more in depth expectations of all participants, as well as recognising the need for time for people with HD to process the concepts of walk organisation. Recent research has highlighted that people with HD participate in physical activity whilst they cope with the nuances of the condition and this changes over time being either self-regulated or regulated in collaboration with carers [22]. This finding needs to be considered when co-producing services that need to adapt over the progression of a long term condition.

### Strengths and Limitations of the Study

This study included a range of relevant stakeholders in the co-production of a walking group for people with HD. Co-production was limited by the small number of people with HD who participated in co-planning and co-execution stages which resulted in the group not continuing when support from the research team was withdrawn. The findings were limited, as data saturation was not reached due to the small sample size.

### Recommendations

Walking was perceived as enjoyable and beneficial and should be considered by healthcare professionals as a way to maintain physical activity in people with HD. It is recommended that further research is undertaken to explore adaptation of models of co-production for people with HD and other neurodegenerative conditions that are cognisant of the consequences of the disease pathology on co-planning and co-execution. These adapted models should also integrate the concepts of self and collaborative regulation that have been identified as a means by which people with HD participate in physical activity.

## Conclusions

Existing evidence demonstrates that exercise is safe, feasible and beneficial for people with HD, yet physical activity levels are low. A supported walking group was perceived as enjoyable, beneficial, and motivational for people with HD. Issues related to apathy, diminished planning skills, social stigma and motor problems specific to HD may have limited potential participants joining the study and impacted on the continuation of the walking group.

Regular participation in physical activity requires support for people with HD. This support, which may alter during the progression of the condition, may include that from carers, healthcare providers or non-profit organisations who can motivate, co-ordinate and potentially lead suitable activities that correspond with the needs of the person with HD. Further research is needed to explore appropriate models of co-production for people with HD and other long term complex conditions.

## Figures and Tables

**Table 1:** Living, working status and Life-Space scores for people with HD.

Participant	Living status	Working status	Life-Space Assessment baseline	Life-Space assessment post supported walking group	Change in mobility
**Sally TFC 9, Stage 2**	Independent, alone	Not working	58	64	Increased frequency of mobility within neighbourhood
**George TFC 9, Stage 2**	Independent, alone	Not working	52	72	Increased mobility outside of town
**John TFC 10, Stage 2**	Independent, alone	Not working	100	110	Increased frequency of mobility outside of town
